# The Effect of Intravesical Bacillus Calmette-Guerin Treatment on Sexual Functions and Mental Health in Male and Female Patients Diagnosed with Non-Muscle Invasive Bladder Cancer

**DOI:** 10.5152/tud.2026.25045

**Published:** 2026-07-24

**Authors:** Elife Kettas Dolek, Melih Biyikoglu, Selahittin Cayan, Samet Senel, Murat Bozlu, Erdem Akbay

**Affiliations:** 1 Department of Urology, Mersin University School of Medicine, Mersin, Türkiye; 2 Department of Urology, Ankara City Hospital, Ankara, Türkiye

**Keywords:** Non-muscle invasive bladder cancer, intravesical immunotherapy, Bacillus Calmette-Guerin, sexual dysfunction

## Abstract

**Objective:**

The aim of this study is to investigate the effect of intravesical Bacillus Calmette-Guerin (BCG) treatment on sexual functions in females and males diagnosed with non-muscle invasive bladder cancer (NMIBC).

**Methods:**

The study included 87 patients, who presented to the authors’ clinic, were diagnosed with NMIBC, and underwent intravesical BCG immunotherapy. All participants were interviewed thrice: before the first dose, after the third dose, and 4 weeks after the sixth dose of BCG.The Demographic Information Form, the Beck Depression Inventory (BDI), the International Index of Erectile Function (IIEF-5) (in males), and the Female Sexual Function Index (FSFI) (in females) were used in all 3 sessions.

**Results:**

Beck Depression Inventory scores of both males(4.74 ± 4.47, 10.22 ± 6.98, and 5.78 ± 4.96, respectively) and females(9.11 ± 5.33, 15.06 ± 5.3, and 9.67 ± 6.61) significantly increased after the third dosebut decreased 4 weeks after the sixth dose of BCG (*P *< .001). In addition, it was observed that the IIEF-5 scores for males(19.7 ± 6.3, 8.24 ± 8.03, and 17.9 ± 7.11, respectively) and the FSFI scores for females(13.03 ± 8.47, 4.83 ± 6.28, and 11.66 ± 9.04 respectively) decreased after the third dosebut increased 4 weeks after the sixth dose of BCG (*P *< .001).

**Conclusion:**

Repeated intravesical BCG treatments for patients with NMIBC may lead to sexual dysfunction (SD) and mental health. In this study, the peak SD was observed during the treatment process among male and female patients diagnosed with NMIBC who initiated a 6-week BCG treatment. In addition, both men and women were found to be mildly depressed during treatment. These findings tended to improve approximately 4 weeks after the end of the treatment.

**Main Points**

- Approximately 75% of patients presenting with a diagnosis of bladder cancer have non-muscle invasive bladder cancer (NMIBC).
- Non-muscle invasive bladder cancer reduces the quality of life due to frequent recurrences, surveillance cystoscopies, and intravesical BCG treatments.
- Sexual dysfunction and mental health were observed during the treatment process among male and female patients diagnosed with NMIBC who initiated a 6-week BCG treatment

## Introduction

Bladder cancer (BC), ranking tenth in incidence among all cancers worldwide, holds the 13th position in cancer-related deaths. Approximately, 75% of patients presenting with a diagnosis of BC have non-muscle invasive bladder cancer (NMIBC).^1^ Among NMIBC cases, 70% experience recurrence and 25% progress to muscle invasion.^2^^,^^3^ Non-muscle invasive bladder cancer reduces the quality of life due to frequent recurrences, surveillance cystoscopies, and intravesical treatments.^4^^-^^8^

Intravesical immunotherapies have demonstrated positive outcomes in preventing the recurrence and progression of NMIBC.^9^ Bacillus Calmette-Guerin (BCG) is the most effective intravesical agent for NMIBC.^10^ Bacillus Calmette-Guerin has been demonstrated to delay cancer progression, reduce the need for radical cystectomy, and increase overall survival.^11^ In addition, over 50% of patients experience treatment-related side effects.^9^ Local side effects occur due to inflammation caused by intravesical administration, while systemic complications arise due to BCG infection.^12^ Sexual dysfunction (SD) is a common condition after BCG treatment and is often overlooked.^13^ The prevalence of female SD is 52.5%,^14^ and the ED prevalence of males (≥40 years) is 33%^15^ in the Turkish population. Since BC is more prevalent in males, there is limited research and data on SD in females undergoing BCG treatment for NMIBC. In addition, recent data indicate that more than 31% of females are affected by SD, including decreased vaginal lubrication and arousal.^16^ Most studies examining sexual functions in BC focus on the impact of radical cystectomy and urinary diversion on muscle-invasive bladder cancer, and there are few studies evaluating sexual functions among patients with NMIBC.^13^^,^^16^^,^^17^ However, NMIBC constitutes approximately 75% of BC cases, and these patients undergo transurethral surveillance cystoscopies and various intravesical treatments that may affect sexual functions.^1^^,^^13^

In a study focusing on SD in NMIBC patients, 38.8% of participants reported being sexually inactive, with 60% of sexually active male patients experiencing erectile dysfunction (ED) and 62.5% of female patients reporting vaginal dryness. Participants also expressed concerns about contaminating their partners with the treatment agent and refrained from being sexually active due to fear of transmitting the disease.^16^ Sighinolfi et al^18^ evaluated the effect of intravesical BCG treatment on the sexual functions of males with NMIBC. They reported that all patients experienced SD during BCG treatment, which, although temporary and reversible, caused a high incidence of ED.^18^

Surveillance strategies involving repeated intravesical treatments and cystoscopies for NMIBC have a negative impact on impaired physical function, mental health and quality of life. Intravesical therapy also tends to have an impact on the psychological aspect of life.^19^ The findings of Wei et al^20^ revealed significant decrease in quality of life. Compared to the baseline, owing to the increase in symptoms (pain, frequent urination, and diarrhea) at the sixth-week week post-intravesical treatment review. Abbona et al^21^ have stated that intravesical treatment causes changes in work life and self-esteem in a significant number of patients.^21^ Bacillus Calmette-Guerin immunotherapies are expected to be optimized soon, decreasing its side effects and improving its efficacy.^22^ The aim of this study was to examine the effect of the initial intravesical BCG immunotherapy on sexual functions and mental health in female and male patients diagnosed with NMIBC, which constitute the majority of BC patients.

## Material and Methods

The study included 87 patients, 69 males, and 18 females, who presented to the authors’ clinic, were diagnosed with NMIBC and underwent intravesical BCG immunotherapy between September 1 and December 31, 2022. The participants were selected from among individuals aged 18 years and older who were motivated, cooperative, sexually active, diagnosed with NMIBC, had started intravesical BCG treatment, and had continued BCG treatment uninterrupted for a 6 weeks. The sample size for the study was determined using the G-Power software with an effect size of 0.25, a type I error of 0.05, and a power of 0.80.

Patients meeting the inclusion criteria, consenting to participate in the study, diagnosed with NMIBC, scheduled for a 6-week intravesical BCG induction therapy, and agreed to participate were interviewed face-to-face 3 times (before the first dose, after the third dose, and 4 weeks after the sixth dose of BCG). Clinical data were obtained with the Demographic Information Form (DIF), prospectively. The Beck Depression Inventory (BDI), the International Index of Erectile Function (IIEF-5) (in males), and the Female Sexual Function Index (FSFI) (in females) were used at each of these 3 sessions.

The BDI is a scale assessing characteristic features and symptoms of depression.^23^ The scale consists of 21 items, each identifying a specific behavioral pattern related to depression, and questions with 4 options ranging from 0 to 3 (least to most). The scores of all answers are summed and evaluated as follows: 0-9 points: minimal, 10-16 points: mild, 17-29 points: moderate, 30-63 points: severe depressive symptoms.

The IIEF evaluates erectile function, orgasmic function, sexual desire, sexual satisfaction, and overall satisfaction.^24^ Men presenting with NMIBC are queried using the Turkish-validated version of the IIEF-5. Erectile status is evaluated based on the obtained score: 5-7 severe, 8-11 moderate, 12-16 mild to moderate, 17-21 mild, and 22-25 normal erectile function.^25^

The FSFI consists of 6 subcategories, including desire, arousal, lubrication, orgasm, satisfaction, and pain, with a total of 19 questions that evaluate female sexual functions.^26^ An FSFI value above 22.7 indicates normal sexual function, while an FSFI value below 22.7 indicates SD. Women presenting with NMIBC are queried using the Turkish-validated version of the FSFI.^27^

Approval for this study was obtained from the University of Mersin School of Medicine Clinical Research Ethics Committee with the decision numbered 2022/564 on August 17, 2022. After explaining the study, an informed consent form was obtained from each participant. The research followed the Helsinki Declaration rules.^28^

Statistical analyses were performed using IBM Statistical Package for the Social Sciences Statistics for Windows, version 22.0 (IBM Corp., Armonk, NY, USA). Descriptive statistics were expressed as minimum, maximum, mean ± SD for continuous variables and frequency and percentage for categorical variables. General Linear Model Repeated Analysis was conducted to compare means of changes in repeated measurements of continuous variables and to identify the effect of various categorical or continuous subjects on these changes. Bivariate correlation analysis was performed to reveal correlations according to periods of indexes. A *P* value less than .05 was considered statistically significant.

## Results

The study included 87 patients: 69 (79.3%) were male and 18 (20.7%) were female The mean age of males was 63.94 ± 8.97 years (40-79), with a mean body mass index (BMI) of 27.44 ± 4.4 kg/m^2^, ranging from 18.52 to 38.87. Females had a mean age of 63.39 ± 6.89 years (51-77), with a mean BMI of 30.36 ± 7.69 kg/m^2^, ranging from 17.65 to 47.87. At the end of the BCG therapy, the majority of patients (77.8%) were still at the pT1 stage, while a smaller proportion (22.2%) progressed to the pT2 stage.

**Figure 1.**
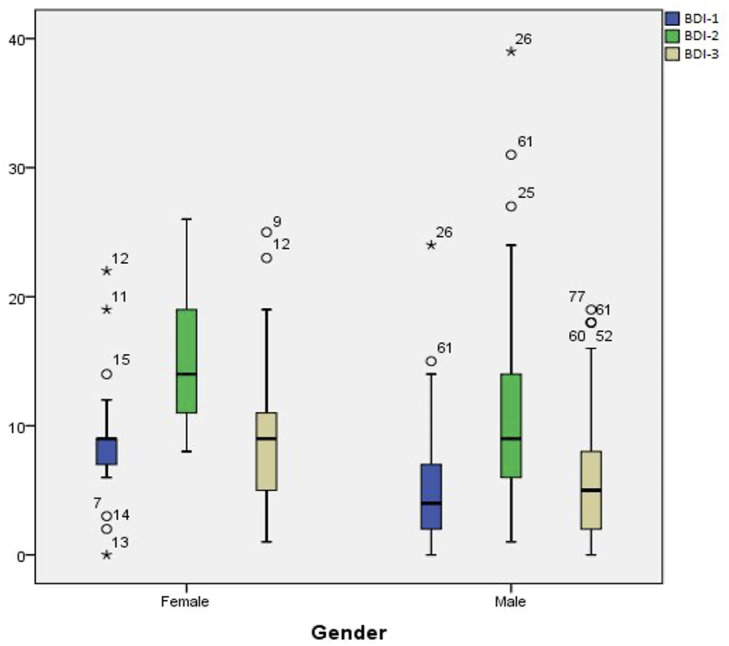
The analysis of the changes in the Beck Depression Inventory over periods in both genders. 1. Before the first dose of BCG; 2. After the third dose of Bacillus Calmette-Guerin; 3. 4 weeks after the sixth dose of Bacillus Calmette-Guerin.

The mean BDI scores of males before the BCG treatment, after the third dose and after the sixth dose were 4.74 ± 4.47, 10.22 ± 6.98, and 5.78 ± 4.96, respectively. The differences between pre-first dose and post-third dose, as well as post-third dose and post-sixth dose, were significant (*P *< .001). Likewise, the mean BDI scores of females before the BCG treatment, after the third dose, and after the sixth dose were 9.11 ± 5.33, 15.06 ± 5.3, and 9.67 ± 6.61, respectively. The differences between pre-first dose and post-third dose, as well as post-third dose and post-sixth dose, were significant (*P* < .001) (Figure 1).

The mean IIEF-5 scores for the same periods were 19.7 ± 6.3, 8.24 ± 8.03, and 17.9 ± 7.11, with significant differences between each pair of time points (*P *<* .*001 for pre-first dose and post-third dose, *P = .*009 for pre-first dose and post-6rd dose, *P *<* .*001 for post-third dose and post-sixth dose). Furthermore, the mean FSFI scores for the same periods were 13.03 ± 8.47, 4.83 ± 6.28, and 11.66 ± 9.04, with significant differences between pre-first dose and post-third dose, as well as post-third dose and post-sixth dose (*P < .*001 for pre-first dose and post-third dose, *P = .*004 for post-third dose and post-sixth dose). The analysis of all subparameters of the FSFI is shown in Table 1. Other demographic and clinical data of the patients are shown in Table 2.

**Figure 2:**
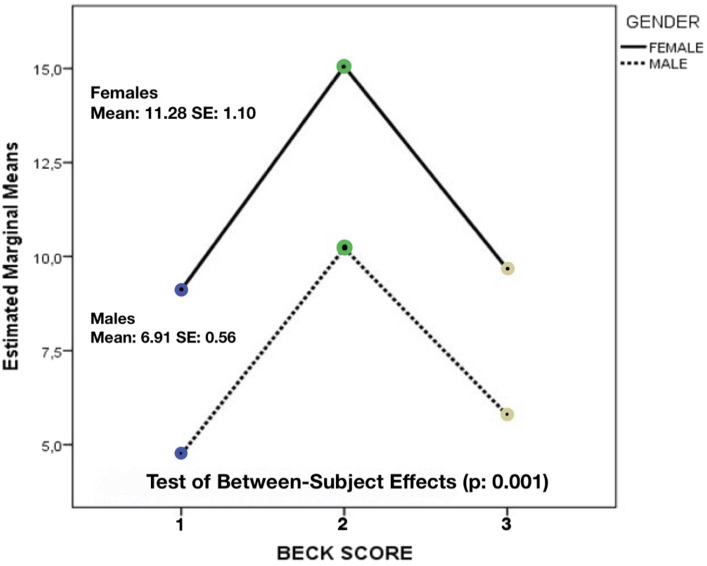
The estimated marginal means of Beck Depression Inventory in General Linear Model Repeated Measures in both genders. SE, standard error.

The mean BDI scores of all 3 periods (before the first dose, after the third dose, and after the sixth dose of BCG) were 6.91 ± 0.56 for males and 11.27 ± 1.10 for females. The mean BDI score of females was significantly higher than males (*P = .*001). In addition, the percentage changes in mean BDI scores across all 3 time periods were similar for both genders (*P* > .05 for pre-first dose and post-third dose, *P* > .05 for pre-first dose and post-sixth dose, *P *> .05 for post-third dose and post-sixth) (Table 3 and Figure 2).

**Figure 3.**
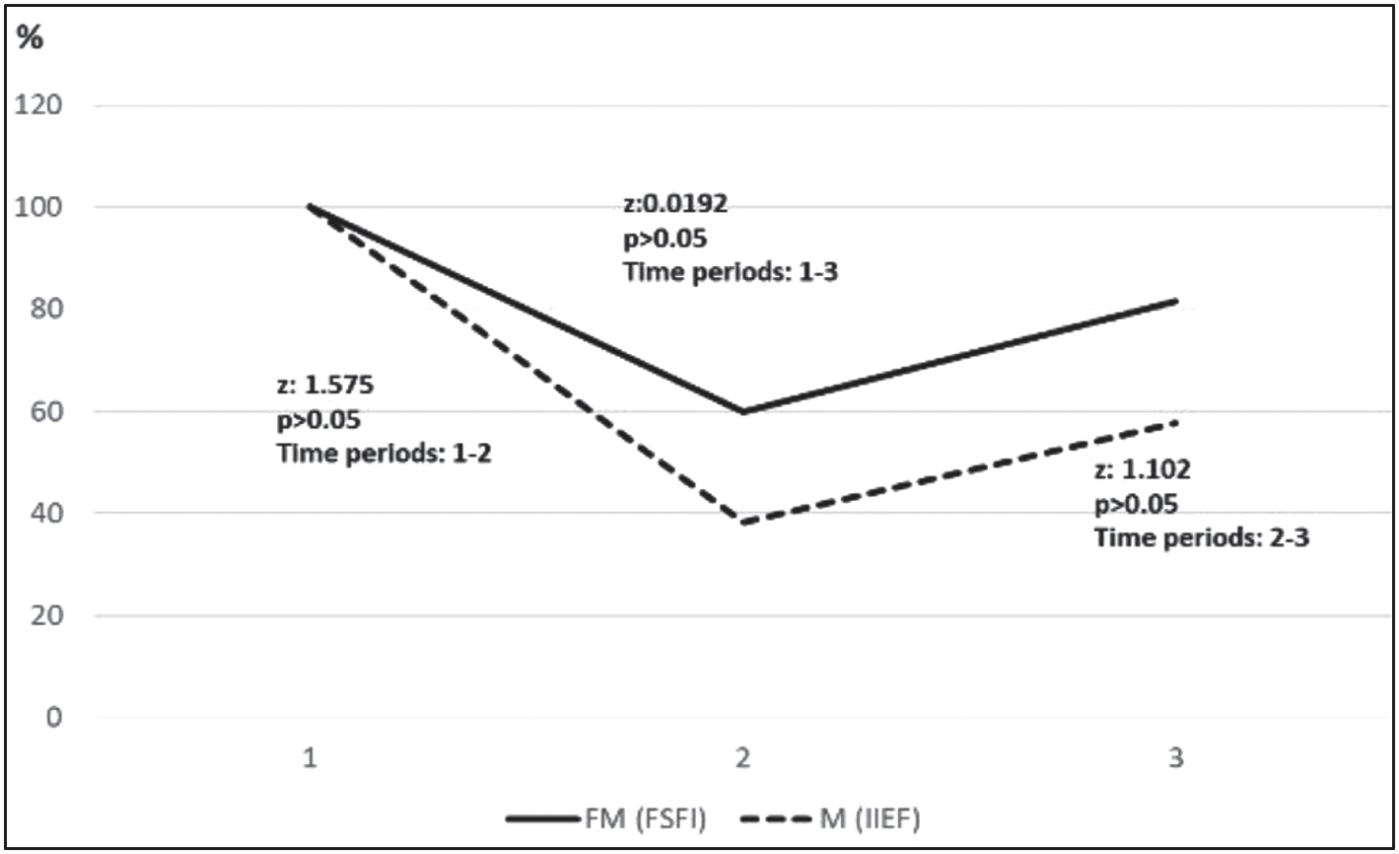
The mean percentage changes in the sexual scales over periods in both genders. FM, females; FSFI, female sexual function index; IIEF, international index of erectile function; M, males.

The percentage changes in mean IIEF-5 scores for males, and FSFI scores for females were also similar across all 3 time periods (*P *> .05 for pre-first dose and post-third dose, *P *> .05 for pre-first dose and post-sixth dose, *P *> .05 for post-third dose and post-sixth) (Table 3 and Figure 3). The patients of both genders were divided into subgroups based on different characteristics such as age, BMI, education level, smoking status, chronic diseases, and related drug use. The mean BDI, IIEF-5, and FSFI scores and the percentage changes in these scores across all 3 periods were compared.

Table 4 includes the analysis of the changes in BDI over time periods based on different subgroups in both genders. The mean BDI scores of the patients with a university graduate level were significantly higher than those with a primary or high school graduate level (*P = .*046 for males, *P = .*037 for females). In addition, there was no correlation between the percentage changes in mean BDI scores across all 3 time periods and education level for both genders (*P = .*601 for males, *P = .*889 for females).

Table 5 includes the analysis of the changes in IIEF-5 for men and FSFI for women over time periods based on different subgroups. For males, those using regular medication due to chronic diseases had a lower mean IIEF-5 score (*P = .*037). However, there was no correlation between the percentage changes in the mean IIEF-5 scores across all 3 periods and regular medication use for chronic diseases (*P = .*997). Nevertheless, there was no correlation between age and mean IIEF-5 score for males (*P = .*249) and between age and mean FSFI score for females (*P = .*423). In addition, there was a positive correlation between the percentage changes in mean IIEF-5 scores across all 3 periods and age for males (*P = .*009).

## Discussion

### Impact on Mental Health

It has been stated that, following the initial diagnosis of NMIBC, patients experience a decrease in quality of life. The reasons for this decline include the burden of frequent invasive follow-ups, the strain and unpleasant side effects of BCG treatment, as well as psychological issues such as anxiety, depression, and uncertainty.^29^ Intravesical therapy has been reported to have a significant impact on patients’ quality of life, leading to challenges such as increased depression and emotional coping difficulties.^5^^,^^30^ Indeed, in this study, side effects related to intravesical therapies such as dysuria, hematuria, and hematospermia were recorded during BCG treatment. This suggests that such symptoms may contribute to psychological problems and sexual issues in patients.

In a study, patients diagnosed with NMIBC receiving BCG therapy experienced declining overall health and quality of life. They also noted significant impacts on patients’ domesticities and social functions.^8^ Bolat et al^7^ reported that female and male patients who followed after the diagnosis of NMIBC were mildly depressed at the end of the first year. In this study, both males and females showed BDI score averages consistent with mild depression midway through BCG treatment, but these scores decreased and returned to normal 4 weeks after the sixth dose. Undesirable symptoms such as pollakiuria, dysuria, or hematuria are seen in patients, especially at the beginning of intravesical BCG therapy, which may have negative effects on the mental health of patients. In addition, the isolation from family and work activities during the treatment may contribute to social isolation, potentially affecting patients’ psychological well-being.

Furthermore, in a study evaluating patients’ quality of life diagnosed with BC, lower urinary tract symptoms were reported more frequent in patients receiving intravesical immunotherapy following transurethral resection. Still, the psychological status of patients receiving intravesical therapy following resection was better compared to patients who underwent transurethral resection alone.^30^ In this study, the authors reported that female patients had significantly higher BDI scores than male patients. However, the percentage changes in BDI score averages were similar for both genders across all 3 periods. In contrast to the authors’ results, Schmidt et al^31^ reported that the depression experienced by patients with NMIBC during the treatment process was more severe than that of normal individuals, with there was no difference between genders.

When comparing the mean BDI scores and their percentage changes across 3 time periods with the demographic characteristics of female and male patients, the authors found that female and male patients with higher education levels had higher BDI scores. Thus, individuals with a higher education level seem to be more affected by the negative effects of intravesical BCG treatment on mental health. In addition, individuals with higher education levels may comprehend and respond to the inventory more accurately, reflecting their reality more accurately. Tapple et al^32^ reported that the BDI requires a higher reading ability than most depression inventories. On the other hand, this situation can be explained by individuals with a higher education level having a more heightened self-awareness, a component of emotional intelligence. Srivastava et al^33^ reported that emotional intelligence plays a significant role in academic success.

From a different perspective, a higher education level may protect against depression. Indeed, studies have reported higher depression rates in individuals with lower education levels compared to those with higher education levels.^34^ Individuals with a higher education level may have a better overall quality of life than those with a lower education level. As a result, they may be able to handle the negative effects of BCG treatment on their life more effectively. In fact, in a study evaluating the quality of life of patients with NMIBC, it was reported that depressive symptoms were higher in patients with lower life quality.^2^ Patients with NMIBC face the potential psychological burden of frequent surveillance cystoscopies and possible intravesical treatments throughout their lives, which may harm their quality of life.

### Impact on Sexual Life

Local and radical treatments of BC in both men and women may directly affect sexual functions, leading to decreased sexual desire or variability in the sexual response cycle, defined as changes in sexual interest and behavior. Sexual functions have increasingly become a component of assessing the quality of life, especially in evaluating genitourinary malignancies.^35^ Data on cancer-induced SD in patients with BC are limited, with most studies focusing on radical cystectomy patients.^35^ Intravesical BCG therapy, a standard treatment for intermediate and high-risk NMIBC patients after transurethral resection,^4^ may lead to annoying symptoms such as dysuria, painful ejaculation, ED, and concerns about contamination between partners.^16^^,^^18^ In this study, the authors reported mild SD in men before the first dose and after the sixth dose of BCG (significantly more severe after the sixth dose than before the treatment); moderate SD was observed after the third dose.

In women, SD was identified in all 3 periods (significantly more severe after the third dose than in other periods). In addition, both in men and women, the lowest average sexual function scale score was observed after the third dose of BCG treatment. Furthermore, the change percentages in IIEF-5 scores for men and FSFI scores for women were similar across the 3 periods. A study evaluating the effect of BCG treatment on erectile functions reported that all patients described SD during BCG treatment, especially experiencing ED within the first 36-48 hours after BCG application.^18^ The authors observed the peak SD in men after the third treatment dose. Although sexual functions did not return to their pre-treatment status after the completion of 6 doses of BCG treatment, improvement was observed. That may indicate that the negative effect of BCG treatment on male sexual functions may be temporary and reversible.

A study evaluating the quality of life and sexual functions of patients who underwent intravesical BCG treatment reported mild to moderate ED in men and SD in women at the end of 1 year. In addition, 24% of patients reported no interest in sexuality, 54% did not feel comfortable with sexual intimacy with their partners, 20% were not sexually satisfied, and 57% were concerned that the medication could harm their partner during sexual intercourse.^7^ Another study on the sexual functions of patients with NMIBC revealed that 38.8% of patients were not sexually active, 23.2% were concerned about transmitting the medication to their partners, and sexually active men reported a 60% incidence of ED, women reported 62.5% vaginal dryness.^16^ Thus, intravesical BCG therapy appears to harm sexual intercourse between partners.

Jung et al^5^ reported that men receiving BCG treatment had better sexual functions and pleasure than women, but they also had a higher level of contamination concern. Another 10-year follow-up study evaluating sexual functions after the diagnosis of BC found that a significant proportion of sexually active men and women reported SD, with women showing less interest in sexuality and being less sexually active compared to men. Likewise, when treatment-related sexual functions were examined, patients who underwent radical surgery were reported to be less sexually active than those who received intravesical treatment.^35^ Literature indicates significantly higher rates of SD in women diagnosed with BC compared to men. The authors observed SD in women at all 3 periods, with the peak at the midpoint of the treatment.

The relationships between IIEF-5 scores for men and FSFI scores for women, along with their changes across the 3 periods, were evaluated in relation to clinical characteristics, including age, BMI, education level, smoking, and medication use for chronic diseases. It was found that male patients using medications for chronic diseases had lower IIEF-5 scores. The etiology of ED is generally multifactorial, such as age, psychological conditions, lifestyle habits, concomitant diseases, and medications used in the treatment of these diseases.^36^ Despite various side effects of drugs, ED is the least discussed one, estimated to occur in approximately 30% of patients due to medications.^37^ Multiple agents, including antihypertensives (such as beta-blockers), antidepressants, antipsychotics, antiepileptics, and H_2_ receptor antagonists, are reported to cause ED.^36^ However, the authors’ results showed no significant relationship between age and IIEF-5 scores in male patients. Still, there was a positive correlation between age and the change in the average IIEF-5 score over the 3 periods. In a study evaluating ED during intravesical BCG treatment, no relationship was found between age and ED.^18^

### Strengths and Limitations

This study has several limitations, including the short follow-up interval, not using a quality-of-life scale, the study being conducted at a single center, and low number of female patients.

Nevertheless, the data obtained contribute to the literature for assessing the effects of intravesical BCG treatment on the sexual functions of patients. The authors’ sample consists of newly diagnosed patients who were prospectively evaluated at 3 close periods during the treatment, planned for a 6-week intravesical BCG initial treatment, allowing for a sensitive assessment of the therapy on mental health and sexual functions. These features constitute the originality of this study.

Repetitive procedures such as intravesical BCG treatment may have negative effects on the quality of life. In this study, the peak SD and depressive symptoms were observed in the middle of the treatment process among male and female patients, diagnosed with NMIBC who initiated a 6-week BCG treatment. These findings tended to improve approximately 4 weeks after the conclusion of the treatment. This study emphasizes the importance of clinicians counseling patients with NMIBC on sexual functions as a part of the quality of life through a multidisciplinary approach. This approach will prepare patients for possible challenges during the treatment.

## Figures and Tables

**Table 1.** The Analysis of the Changes in the Female Sexual Function Index Subcategories over Periods in Females

Parameters	N	Mean ± SD [min-max]	*P* ^**^	*P* ^***^
Desire-1	18	2.267 ± 1.02 [1.2-3.6]	.000	1-2: **.003** 1-3: .868 2-3: **.001**
Desire-2	18	1.40 ± 0.82 [0-3.0]		
Desire-3	18	2.23 ± 0.94 [1.2-3.6]		
Sexual arousal-1	18	1.73 ± 1.34 [0-3.6]	.000	1-2: **.000** 1-3: .906 2-3: **.000**
Sexual arousal-2	18	0.45 ± 1.03 [0-3.3]		
Sexual arousal-3	18	1.70 ± 1.69 [0-4.2]		
Lubrication-1	18	2.36 ± 1.78 [0-4.8]	.000	1-2: **.000** 1-3: .051 2-3: **.000**
Lubrication-2	18	0.58 ± 1.34 [0-3.6]		
Lubrication-3	18	1.83 ± 1.80 [0-4.5]		
Orgasm-1	18	2.00 ± 1.62 [0-4.8]	.000	1-2: **.000** 1-3: .493 2-3: **.004**
Orgasm-2	18	0.49 ± 1.13 [0-3.2]		
Orgasm-3	18	1.78 ± 1.76 [0-4.4]		
Satisfaction-1	18	2.20 ± 1.24 [0.8-4.8]	.001	1-2: **.001** 1-3: .359 2-3: **.013**
Satisfaction-2	18	1.07 ± 0.86 [0-3.6]		
Satisfaction-3	18	1.98 ± 1.52 [0-4.8]		
Pain-1	18	2.47 ± 1.90 [0-5.6]	.003	1-2: **.002** 1-3: .076 2-3: **.012**
Pain-2	18	0.80 ± 1.48 [0-4.4]		
Pain-3	18	2.11 ± 1.73 [0-4.4]		

1. Before the first dose of BCG; 2. After the third dose of BCG; 3. 4 weeks after the sixth dose of BCG. Bold values indicate statistically significant differences.

**Table 2.** The Frequency of Some Categorized Variables in Patients

Variable		Frequency (Female)	%	Frequency (Male)	%	*P* ^**^
Education^*^	1	2	11.1	1	1.4	.070
	2	14	77.8	50	72.5	
	3	2	11.1	18	26.1	
Smoking	No	13	72.2	39	56.5	.226
	Yes	5	27.8	30	43.5	
Chronic disease	No	7	38.9	31	44.9	.646
	Yes	11	61.1	38	55.1	
Drug use	No	6	33.3	26	37.7	.733
	Yes	12	66.7	43	62.3	
Operation status	No	9	50.0	36	52.2	.869
	Yes	9	50.0	33	47.8	
Pregnancy	0	2	11.2			
	1-2	8	44.4			
	>2	8	44.4			
Delivery method	Vaginal	14	87.5			
	Cesarean	2	12.5			

*1. Primary School; 2. High School; 3. University.

**Chi-square test.

**Table 3.** The Comparisons of Mean Percentage Change Over Periods Between Both Genders for Beck Depression Inventory and International Index of Erectile Function or Female Sexual Function Index

Score	Gender	Time Period	n	Mean % change	SD	*P*
BDI	Female	1-2	18	+38.28	29.40	.264^*^
	Male	1-2	69	+50.32	42.46	
	Female	2-3	18	−39.72	25.92	.921^*^
	Male	2-3	69	−40.69	39.00	
	Female	1-3	18	31.78	124.41	.555^*^
	Male	1-3	69	62.56	203.60	
FSFI (FM) IIEF (M)	Female	1-2	18	−400.39	448.54	>.05^**^
	Male	1-2	69	−617.28	732.80	
	Female	2-3	18	+359.26	479.30	>.05^**^
	Male	2-3	69	+508.44	617.82	
	Female	1-3	18	−6.46	32.52	>.05^**^
	Male	1-3	69	−6.79	127.04	

BDI, Beck Depression Inventory; FSFI, International Index of Erectile Function; IIEF, International Index of Erectile Function. *Student’s *t*-test. **Z test.

**Table 4.** The Results of GLM Repeated-Measures Analysis for Beck Depression Inventory

Gender	Within-Subject Variable (BDI—Time Periods:3)	Tests of Within-Subjects Effects (*P*)	Subject Factors	Tests of Between-Subjects Effects (*P*)
Male	BDI BDI*Age (≥66, <66)^a^	**.000**	Age	.956
		.065		
	BDI BDI*BMI (≥30, <30)	**.000**	BMI	.457
		.608		
	BDI BDI*Education (1, 2, 3)^b^	**.000**	Education	**.046**
		.601		
	BDI BDI*Smoking (Yes, No)	**.000**	Smoking	.139
		**.048**		
	BDI BDI*Chronic disease (Yes, No)	**.000**	Chronic disease	.227
		**.022**		
	BDI BDI*Drug use (Yes, No)	**.000**	Drug use	.377
		**.041**		
	BDI BDI*Operation status (Yes, No)	**.000**	Operation status	.218
		.843		
Female	BDI BDI*Age (≥64, <64)^a^	**.000**	Age	.200
		.525		
	BDI BDI*BMI (≥30, <30)	**.000**	BMI	.220
		**.013**		
	BDI BDI*Education (1, 2, 3)^b^	**.003**	Education	**.037**
		.889		
	BDI BDI*Smoking (Yes, No)	.000	Smoking	.245
		.114		
	BDI BDI*Chronic disease (Yes, No)	**.000**	Chronic disease	.220
		.958		
	BDI BDI*Drug use (Yes, No)	**.000**	Drug use	.282
		.935		
	BDI BDI*Operation status (Yes, No)	**.000**	Operation status	.507
		.208		
	BDI BDI*Pregnancy (0, 1, 2)^c^	**.000**	Pregnancy	.123
		.135		
	BDI BDI*Delivery method (1, 2)^d^	**.000**	Delivery method	.531
		**.000**		

BDI, Beck Depression Inventory; GLM, General Linear Model.

^a^Median value.

^b^1. Primary School; 2. High School; 3. University.

^c^0 (0); 1. (1,2); 2. (3,3+).

^d^1. Vaginal; 2. Cesarean.

**Table 5.** The Results of GLM Repeated-Measures Analysis for International Index of Erectile Function-5 and Female Sexual Function Index

Gender	Within-Subject Variable (IIEF-5, FSFI—Time Periods:3)	Tests of Within-Subjects Effects (*P*)	Subject Factors	Tests of Between-Subjects Effects (*P*)
Male	IIEF IIEF*Age (≥66, <66)^a^	**.000**	Age	.249
		**.009**		
	IIEF IIEF*BMI (≥30, <30)	**.000**	BMI	.141
		.498		
	IIEF IIEF*Education (1, 2, 3)^b^	**.000**	Education	.561
		.611		
	IIEF IIEF*Smoking (Yes, No)	**.000**	Smoking	.582
		.355		
	IIEF IIEF*Chronic disease (Yes, No)	**.000**	Chronic disease	.396
		.309		
	IIEF IIEF*Drug use (Yes, No)	**.000**	Drug use	**.037**
		.997		
	IIEF IIEF*Operation status (Yes, No)	**.000**	Operation status	.448
		.198		
Female	FSFI FSFI*Age (≥66, <66)^a^	**.000**	Age	.423
		.901		
	FSFI FSFI*BMI (≥30, <30)	**.001**	BMI	.280
		.091		
	FSFI FSFI*Education (1, 2, 3)^b^	.069	Education	.131
		.588		
	FSFI FSFI*Smoking (Yes, No)	**.000**	Smoking	.728
		.643		
	FSFI FSFI*Chronic disease (Yes, No)	**.000**	Chronic disease	.756
		.701		
	FSFI FSFI*Drug use (Yes, No)	**.001**	Drug use	.594
		.602		
	FSFI FSFI*Operation status (Yes, No)	**.000**	Operation status	.182
		.761		
	FSFI FSFI*Pregnancy (0, 1, 2)^c^	**.000**	Pregnancy	.743
		.102		
	FSFI FSFI*Delivery method (1, 2)^d^	**.001**	Delivery method	.470
		.106		

BDI, Beck Depression Inventory; FSFI, Female Sexual Function Index; IIEF, International Index of Erectile Function.

^a^Median value.

^b^1. Primary School; 2. High School; 3. University.

^c^0. (0); 1. (1,2); 2. (3,3+).

^d^1. Vaginal; 2. Cesarean.

## Data Availability

The data that support the findings of this study are available on request from the corresponding author.
